# Supplementary material for: Structural Modeling of Chromatin Integrates Genome Features and Reveals Chromosome Folding Principle
Source: Sci Rep. 2017 Jun 6;7:2818. doi: 10.1038/s41598-017-02923-6 (PMC5460185; doi:10.1038/s41598-017-02923-6)
Supplement: Supplementary file 1 — SI [file 41598_2017_2923_MOESM1_ESM.pdf]

SUPPLEMENTAL INFORMATION

Structural Modeling of Chromatin Integrates Genome Features and Reveals Chromosome Folding Principle

Wen Jun Xie[1,2], Luming Meng[1,2], Sirui Liu[1,2], Ling Zhang[1], Xiaoxia Cai[1], Yi Qin Gao[1,*]

[1] Beijing National Laboratory for Molecular Sciences, College of Chemistry and Molecular Engineering, and Biodynamic Optical Imaging Center, Peking University, Beijing 100871, China

[2] Co-first author

*Correspondence: gaoyq@pku.edu.cn (Y.Q.G)

SI text

1. Characterization of the modeled structure ensemble

By spectral clustering, the 300 conformations were grouped into two sets based on their pair-wise RMSD similarity (Fig. S3). Chromosome structures within the same set are similar to each other and neither set has dominant structures. Conformations from the two sets are found to be mirror-images of each other, similar as another modeling study on Hox gene cluster [1]. If we plot the RMSD matrix between conformations from the first group and the mirror-images of conformations from the second, the gap between two groups disappears and all the conformations become similar. Among all the 300 conformations (the conformation in the second group were reflected to their mirror images), we chose the cluster centroid as the representative for 3D visualization in the manuscript.

In the modeled conformation, the IMR90 chromosome can be viewed as a packing of multiple strands. Due to the segregation of A/B compartments in 3D space, in most cases one strand can be divided into two halves, each belonging to one type of compartment.

2. Knot invariant of the modeled structure ensembles

To characterize the topological structure of our models, we calculated its knot invariant following the idea of the Shrink-On-No-Overlaps algorithm [2]. The distribution of knot invariant is shown in Fig. S5.

We converted each chromosome to a closed rope and minimized its contour length while preserving its topological structure. Following Zhang *et al.*[3], the knot invariant was defined as the ratio between the minimal contour length and the width of the rope. If the chromosome adopts a knot-free structure, the rope will be optimized to a ring and the knot invariant will be close to π. High knot invariants indicate complicated topological structures.

In calculating the knot invariant, we first connected two ends of the chromosome to get a closed structure. To avoid collisions between the new connection and the modeled structure, both ends of the polymer chain were extended along the vector pointing from its mass center to the corresponding end until the distances between the two new ends and the mass center reach $10R_g$, where R_g is the radius of gyration of the modeled structure. The two new ends were then connected by an arc with a radius of $10R_g$. The closed polymer chain was then converted to a rope of consecutive beads along the chain sequence with the bead radius r_b set as 500 nm and the linker distance d_b of two consecutive beads set as 200 nm.

We then minimized the rope length with a 1000-step iteration with its topological structure maintained. For each step, we shrank the rope with a factor of 0.8. Then we reduced the total length of the rope by successively replacing each bent segment along the sequence with a straight line of beads, as long as the region enclosed by the original and replacing segments had no bead chains going through. To reduce the overlap of segments induced by

shrinking, we performed 100 steps of MD simulations on the shortened rope in each iteration step. The bond energy for neighboring beads was written as $E_{bond} = \frac{5}{8} kT(d - d_b)^4$, and a repulsive potential $E_{rep} = \begin{cases} 20000kT(2r_b - d) & \text{if } d < 2r_b \\ 0 & \text{else} \end{cases}$ was used for any two beads separated by at least 5 beads along the string to avoid spatial overlapping. Here k is the Boltzmann constant, T is the temperature, d is the spatial distance between two beads, r_b and d_b are constants defined earlier. Typically, the contour length of the rope converges after the above procedure is repeated for about 100 times. We performed 1000 steps of iteration to ensure convergence and calculated the knot invariant for each conformation.

3. Reproduction of chromatin loops in modeled structure

We further validated our modeling method by reproducing the chromatin loops identified in experimental Hi-C data [4]. To describe the propensity to form a chromatin loop anchored by polymer beads i and j, we defined a ratio $r_{ij,loop} = \frac{<d_{ij}>}{<d(|i-j|)>}$, where $< d_{ij} >$ is the average distance between beads i and j, and $< d(|i - j|) >$ represents the average distance between all bead pairs with the genomic distance of $|i - j|$. Both averages are over modeled conformations.

As a result, 98.2% of the bead pairs anchoring chromatin loops have $r_{ij,loop}$ values smaller than 1. In contrast, for all bead pairs, only 50% have $r_{ij,loop}$ values smaller than 1. The small values of $r_{ij,loop}$ for chromatin loop

anchors indicates that our modeling method successfully captures the chromatin loops.

4. Estimation of chromosome spatial density

In our coarse-grained model for chromosome, the number of polymer beads around a chosen one in a given volume can be regarded as an estimation of bead spatial density. Here we took into account the beads within a distance of 2μm from the central bead. The average neighboring bead number for compartment A and B are 109 and 160, respectively. Thus, we can conclude that the spatial density obtained from our modeling in compartment A is smaller than compartment B.

We also quantified the spatial density in our modeled type A and type B chromatin and the respective neighboring bead numbers for these two types of chromatin are 115 and 158. Therefore, type A chromatin is looser than type B chromatin, in line with the higher-order chromatin compartments. We also calculated the radius of gyration of our modeled type A and type B chromatin and the respective values are 345μm and 277μm, again suggesting the more compact structure in type B chromatin.

5. Identification of A/B compartments

Principal component analysis was previously used to identify the A/B compartments from Hi-C data [5]. In this work, A/B compartments were

identified by spectral clustering of the Hi-C contact frequency matrix with a 200-kb resolution. We first normalized the contact matrix by dividing each element of the matrix by mean contact probability of all segment pairs of the same genomic distance, and obtained the correlation matrix by calculating the Pearson correlation of every two columns of the normalized contact matrix. The correlation matrix was transformed into the similarity matrix with $S_{ij} = \exp(P_{ij})$, where S_{ij} and P_{ij} are the similarity and Pearson correlation of genomic segments i and j, respectively. Then we performed spectral clustering on the similarity matrix and divided all the chromatin segments into two sets. By calculating the average spatial density of the modeled 3D structure for each set, we assigned the one with the lower density to compartment A, and the other to compartment B.

6. Constructing degree of compartmentalization

With the identification of A/B compartment, an index I_i is defined to describe the degree of compartmentalization for the ith genomic region as $I_i = \log\left(\frac{C_n(i,A)}{C_n(i,B)}\right)$, in which $C_n(i,A) = \frac{\sum_{j \in A} C_{ij}/C(|i-j|)}{N_A}$ is the normalized contact frequency averaged in compartment A, N_A is the total number of genomic regions in compartment A. $C_n(i,B)$ is defined accordingly [6]. For each region, a positive sign of this parameter corresponds to compartment A, and a negative one, to compartment B. A high absolute value of I_i indicates that this genomic region lies in the interior of the particular compartment.

7. PMD and non-PMD identification

PMDs were identified genome-wide using a sliding window approach as described by Lister *et al.* [7] with a window size of 10 kb. We identified a region as PMD if there were at least 10 methylated (methylation level greater than 0) CpG dinucleotides within, of which the average methylation level was less than 0.7. We then merged continuous PMD windows to form longer PMDs. Non-PMDs were defined as the complementary set of PMDs.

8. Comparison between the compactness of PMD and non-PMD

The reference genome in mapping the Hi-C data for IMR90 is b37 (hg19) (GSE63525) [4]. The raw observed contact matrix was firstly normalized using the KR normalization method.

The methylation data for IMR90 from Lister *et al.* [7] were aligned to reference genome hg18. In order to compare the chromosome contact in PMD and non-PMD, we converted the reference genome for methylation data from hg18 to hg19 using UCSC liftOver which is the same as Hi-C data.

We first demonstrated that the majority of PMDs coincide with TADs. PMDs tend to overlap with one TAD (the ratio for overlapping percentage in the range of 90~100% is 0.24) or don't overlap with any TADs (the ratio for overlapping percentage in the range of 90~100% is 0.49) (Fig. S8a). The boundary of PMDs also coincide well with TADs (Fig. S8b).

We calculated the contact probability along genomic distance for PMD and non-PMD (Fig. S9a). The contact probability for PMD decays much slower than non-PMD, suggesting the spatial structure for PMD is more compact than non-PMD.

We also used Hi-C data from different sources to show that our conclusion on the different compactness between PMD and non-PMD is robust. The Hi-C data from Dixon *et al.* [8] have a lower resolution (40 kb) than the above data from Rao *et al.* used in the text (10 kb). The hg18 reference genome was used by Dixon *et al.* Thus we can directly combine this Hi-C data with the above methylation data for IMR90 cell line. The contact probability for PMD and non-PMD in IMR90 were shown in Fig. S9b. Compared to non-PMD, PMD chromosomes have a much slower decrease which is qualitatively the same as that in Fig. S9a.

SI References

1. Acemel RD, Tena JJ, Irastorza-Azcarate I, Marletaz F, Gomez-Marin C, de la Calle-Mustienes E, Bertrand S, Diaz SG, Aldea D, Aury JM, et al. A single three-dimensional chromatin compartment in amphioxus indicates a stepwise evolution of vertebrate Hox bimodal regulation. Nat Genet. 2016; 48:336-341.
2. Pieranski P. In search of ideal knots. in 'Ideal Knots', eds. Stasiak A, Katritch V, Kauffman LH (World Scientific, Singapore). 1998.
3. Zhang B, Wolynes PG. Topology, structures, and energy landscapes of human chromosomes. Proc Natl Acad Sci USA. 2015; 112:6062-6067.
4. Rao SS, Huntley MH, Durand NC, Stamenova EK, Bochkov ID, Robinson JT, Sanborn AL, Machol I, Omer AD, Lander ES, Aiden EL. A 3D map of the human genome at kilobase resolution reveals principles of chromatin looping. Cell. 2014; 159:1665-1680.
5. Lieberman-Aiden E, van Berkum NL, Williams L, Imakaev M, Ragoczy T, Telling A, Amit I, Lajoie BR, Sabo PJ, Dorschner MO, et al. Comprehensive mapping of long-range interactions reveals folding principles of the human genome. Science. 2009; 326:289-293.

6. Dileep V, Ay F, Sima J, Vera DL, Noble WS, Gilbert DM. Topologically associating domains and their long-range contacts are established during early G1 coincident with the establishment of the replication-timing program. Genome Res. 2015; 25:1104-1113.
7. Lister R, Pelizzola M, Dowen RH, Hawkins RD, Hon G, Tonti-Filippini J, Nery JR, Lee L, Ye Z, Ngo QM, et al. Human DNA methylomes at base resolution show widespread epigenomic differences. Nature. 2009; 462:315-322.
8. Dixon JR, Selvaraj S, Yue F, Kim A, Li Y, Shen Y, Hu M, Liu JS, Ren B. Topological domains in mammalian genomes identified by analysis of chromatin interactions. Nature. 2012; 485:376-380.

SI Figures

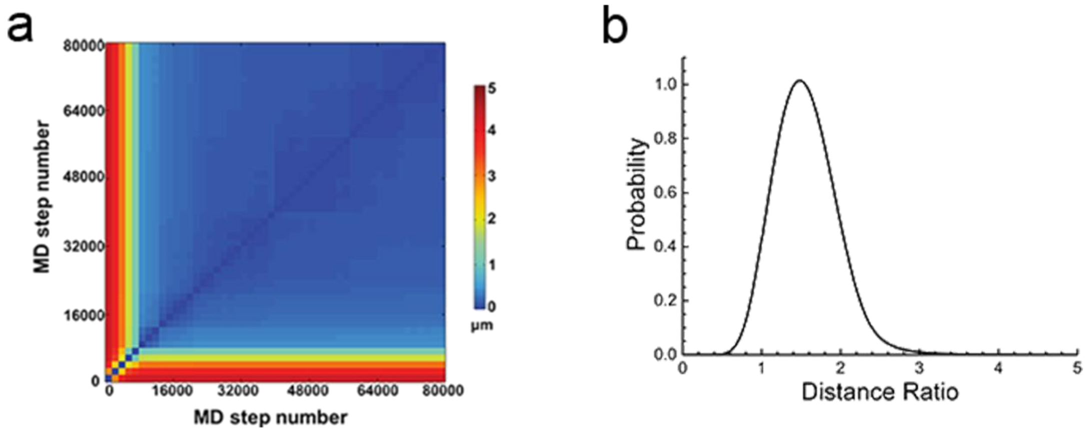

Figure S1. Evaluation of chromosome models for chromosome 1 in IMR90.

(a) The evolution of RMSD of the constructed models with the MD simulation step. The RMSD has converged long before the simulation ends. (b) The distribution of the ratio between modeled and restraint distance.

a

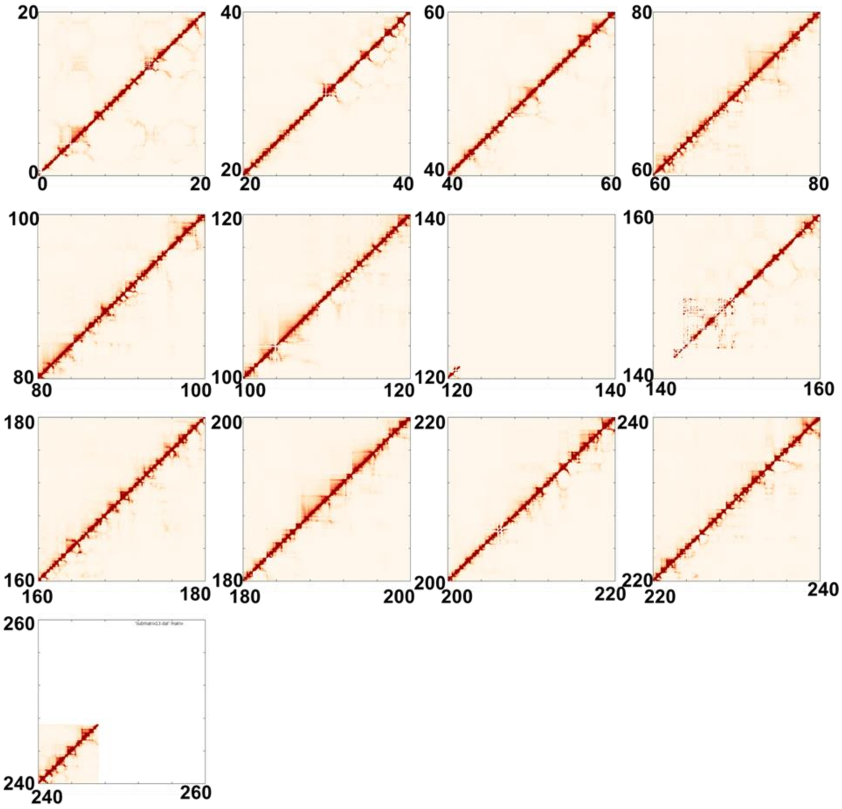

20 0 0 20
40 20 20 40
60 40 40 60
80 60 60 80
100 80 80 100
120 100 100 120
140 120 120 140
160 140 140 160
180 160 160 180
200 180 180 200
220 200 200 220
240 220 220 240
260 240 240 260

b

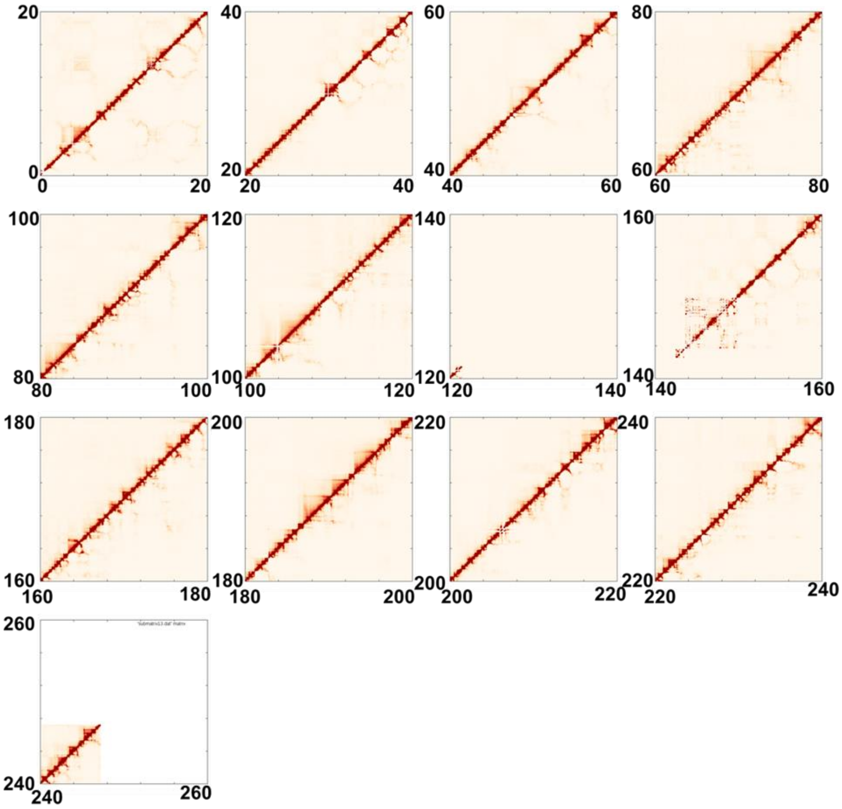

20 0 0 20
40 20 20 40
60 40 40 60
80 60 60 80
100 80 80 100
120 100 100 120
140 120 120 140
160 140 140 160
180 160 160 180
200 180 180 200
220 200 200 220
240 220 220 240
260 240 240 260

Figure S2. Reproduction of Hi-C contact matrix with modeled conformations for chromosome 1 in IMR90.

Experimental Hi-C matrix (upper triangle) and modeled Hi-C matrix (lower triangle) are compared in 20-Mb chromosome segments. For (a), the modeled contact matrix is calculated using 300 models. For (b), only 20 models are used to obtain the modeled contact matrix. Compared with experimental data, our modeled matrix can successfully reproduce the features of the block-wise pattern along the diagonal of the matrix, which has been used to identify TADs. Furthermore, the reproduction of Hi-C matrix can be achieved with only 20 conformations, less than 7% of all the conformations generated and used, showing the convergence of simulated structures.

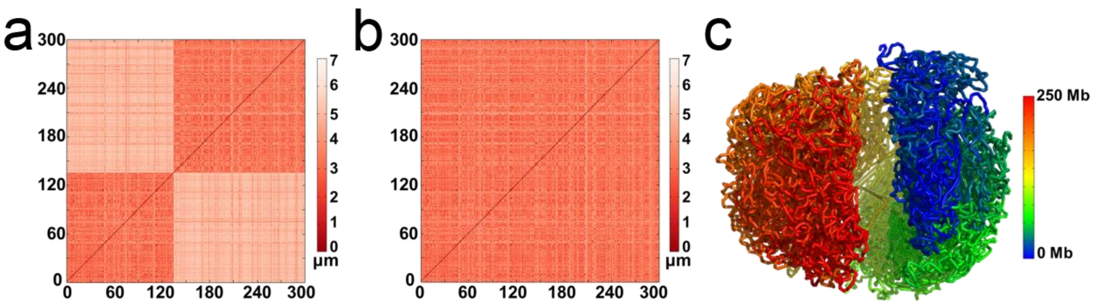

Figure S3. Characterization of the modeled structure ensemble for chromosome 1 in IMR90.

(a) RMSD between any two conformations in the 300 constructed models. Each of the two blocks along the diagonal in the RMSD matrix represents one conformation group. (b) Similar to a while the mirror-images of the second group were used. All conformations show structural similarity. (c) Alignment of five randomly selected modeled conformations.

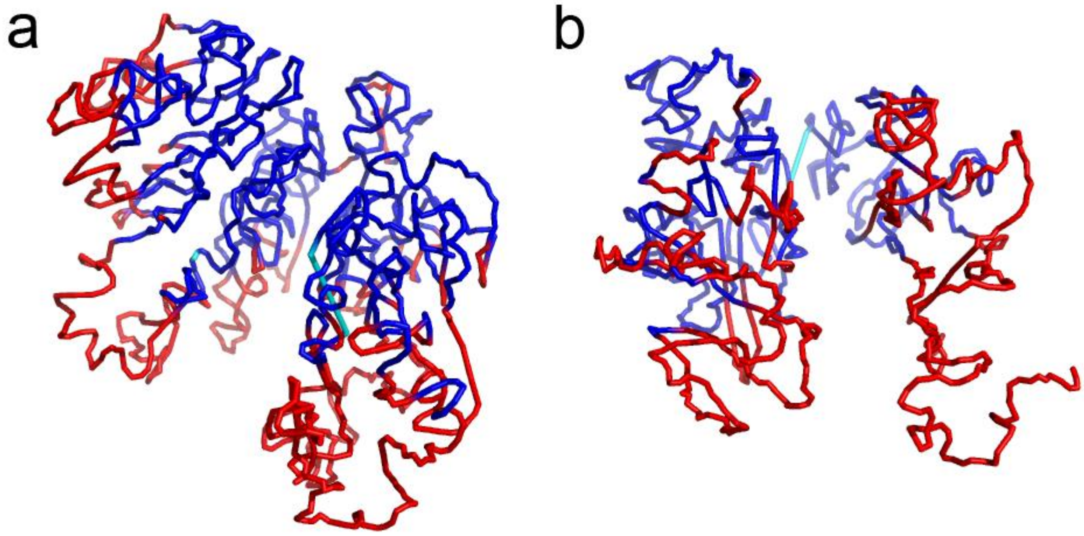

Figure S4. Representative modeled structure for (a) chromosome 18 and (b) chromosome 19 in IMR90.

Chromosome 18 and 19 in IMR90 shows a packed non-spherical configuration. The red and blue color in the structure represents compartment A and B, respectively.

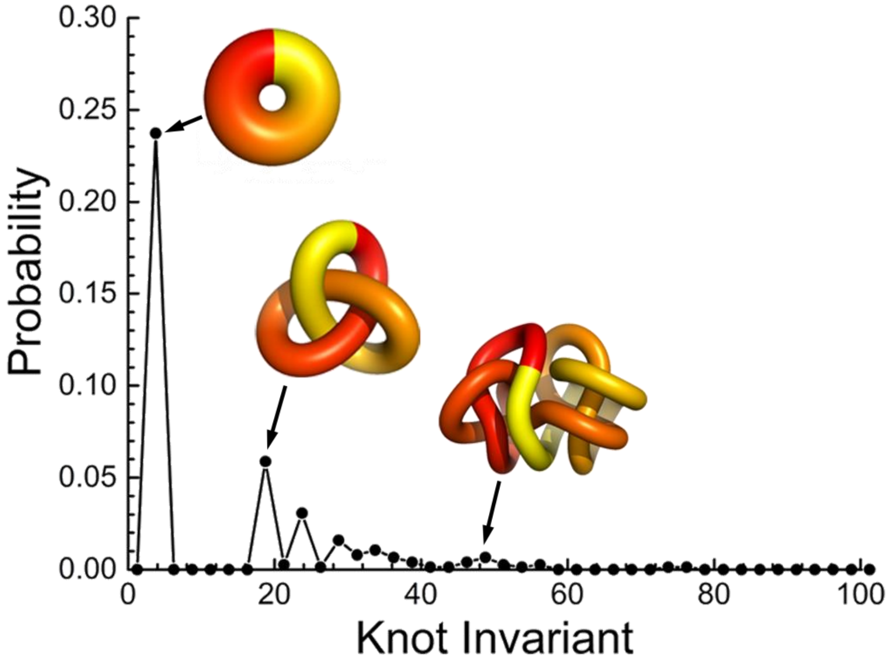

Figure S5. Probability density distribution of the knot invariant for 300 constructed models.

Representative optimized conformations for trivial knot, trefoil knot, and a complex knot structure are shown for visualization. The corresponding knot invariant for each example conformation is pointed to with an arrow. Among all the conformations, 59.3% are optimized to trivial knot, suggesting our conformation ensemble is largely devoid of knot.

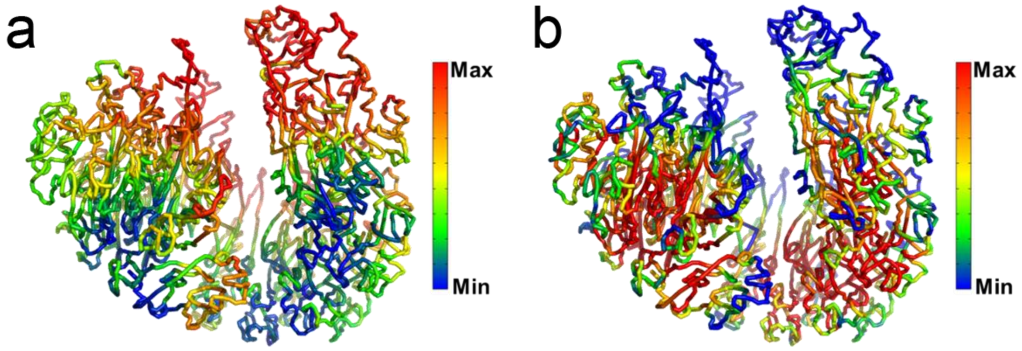

Figure S6. Comparison of the densities between compartments A and B in models for chromosome 1 in IMR90.

Degree of compartmentalization (a) and spatial density in our models (b) are projected on the representative structure.

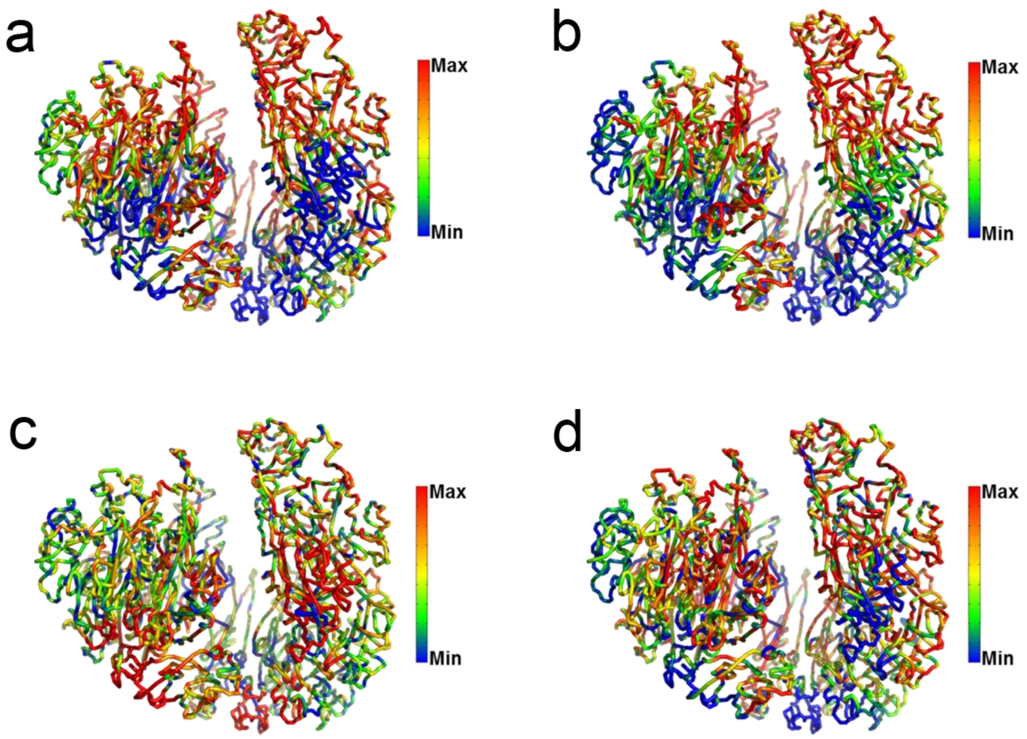

Figure S7. Mapping of histone marks onto the 3D chromatin structure for chromosome 1 in IMR90.

Active histone marks: (a) H3K27ac and (b) H3K36me3. Repressive histone marks: (c) H3K9me3 and (d) H3K27me3.

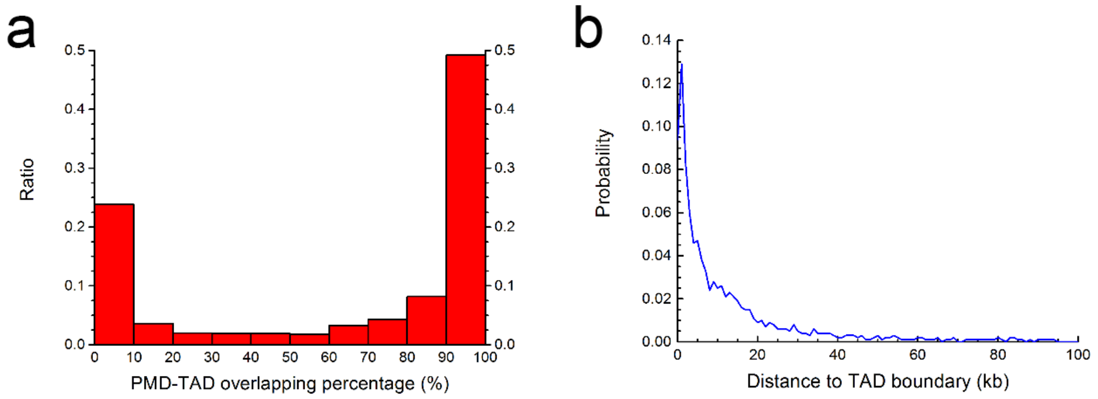

Figure S8. Relation between PMD and TAD.

(a) The overlapping percentage between PMD and TAD for IMR90. (b) The distance between one PMD to its nearest TAD boundary for IMR90.

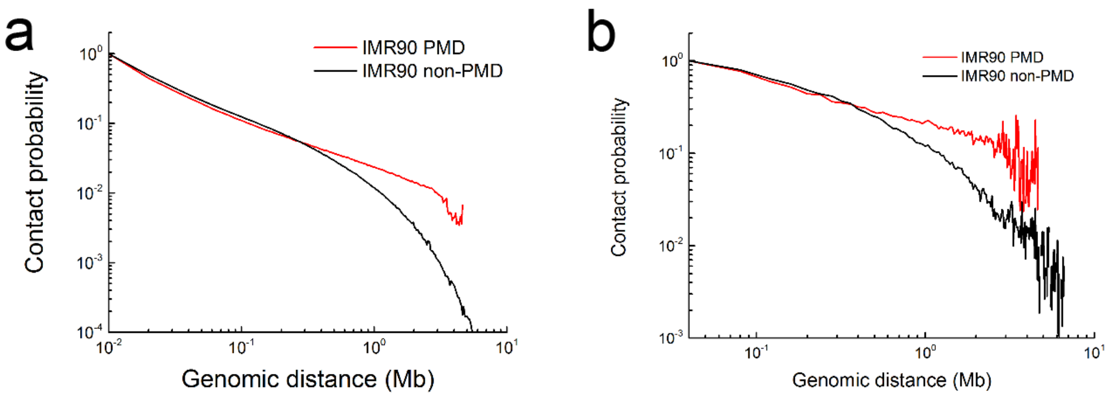

Figure S9. PMD is more compact than non-PMD.

Contact probability derived from the Hi-C data as a function of genomic distance in IMR90: (a) Hi-C obtained from S.S.P. Rao et al., *Cell*, 2014, **159**, 1665 and (b) Hi-C obtained from J. R. Dixon *et al., Nature*, 2012, **485**, 376.
